# When the Gut Tells a Story: Bezoars in a Neglected Autistic Child

**DOI:** 10.7759/cureus.44775

**Published:** 2023-09-06

**Authors:** Saleh A Ba-Shammakh, Eman A Al-Zughali, Salwa M Al-Bustanji

**Affiliations:** 1 Department of General Surgery, The Islamic Hospital, Amman, JOR; 2 Department of Internal Medicine, The Islamic Hospital, Amman, JOR

**Keywords:** rapunzel syndrome, psychiatric disorders, pediatric, ctap, fecal impaction, abdominal pain, autism, trichophagia, trichobezoar, bezoar

## Abstract

This case study delves into the unique presentation of bezoars in a 14-year-old autistic female who exhibited chronic diarrhea and abdominal pain. While trichobezoars, masses formed from ingested hair, are rare, they are predominantly seen in young females and are associated with psychiatric conditions. Through rigorous diagnostic procedures, including a computed tomography imaging of the abdomen and pelvis (CTAP) scan, fecal impaction, and multiple bezoars, including hair and non-biological items, were identified. The background revealed significant neglect, emphasizing the importance of a comprehensive approach that integrates medical, surgical, and psychosocial care.

## Introduction

A bezoar is a mass formed by the accumulation of indigestible materials in the gastrointestinal tract, which can be composed of various substances such as vegetable fibers, human components, hair, medication, or even items like paper, styrofoam, and gloves [1]. Trichobezoars are primarily composed of hair and are frequently observed in younger females, often being a significant consequence of trichophagia, an obsessive-compulsive tendency to consume hair [2]. Trichotillomania, a compelling desire to extract one's hair, is commonly a precursor to trichophagia [3]. Different from other bezoars, trichobezoars aren't typically related to changes in gastrointestinal motility but are more linked with specific psychiatric conditions. These conditions might include post-traumatic stress disorder (PTSD), often resulting from childhood trauma such as neglect or maltreatment, and mood disorders [4-5]. A rare presentation of trichobezoar, where it stretches from the stomach to the small intestine, is called "Rapunzel syndrome". This condition was initially documented in 1968 by Vaughan et al. [6]. Despite the documented surgical procedures for trichobezoars, their psychiatric etiology is not thoroughly explored in the literature [7]. Clinically, these may manifest with symptoms such as abdominal pain, anorexia, or obstruction and, if left untreated, can lead to complications including gastric erosions, ulcerations, or perforation [8].

## Case presentation

A 14-year-old female, known to have autism, came to our attention primarily due to gastrointestinal symptoms. She exhibited signs of the condition since the age of two and was diagnosed with autism at the age of three. Despite this diagnosis, there was no regular follow-up or medical intervention for her condition. As she was non-verbal, her medical history was primarily relayed by family members.

She had been battling diarrhea for approximately six weeks. Initially, it was observed that loose to soft stools passed about four times daily. However, in the two weeks leading up to her hospital admission, the frequency escalated to six to seven times daily. These stools were brown, large in quantity, and lacked any traces of blood or mucus. They were also devoid of any foul odor and were easily flushed. Along with this, she exhibited signs of lower abdominal pain, which the family recognized as she would lean forward and compress her abdomen, suggesting a colicky nature to the pain. Despite these symptoms, there were no reports of difficulties swallowing, vomiting, fever, or any weight or appetite changes.

In terms of her medical history, there were no previous similar episodes, no history of recent travels, or any sick contacts. A general practitioner had previously assessed her symptoms, attributing them to simple gastroenteritis. She was prescribed amoxicillin and clarithromycin, but no improvement was observed post-treatment.

Upon evaluation in our emergency room, her vitals were recorded as follows: blood pressure of 110/75 mmHg, heart rate of 120 bpm (regular), oral temperature of 37°C, respiratory rate of 18 breaths per minute, and oxygen saturation of 96% on room air. Clinically, she appeared to be in pain and displayed irritability. With her autism, she primarily communicated through sounds and frequently fixed her gaze in one direction. A chest examination was unremarkable. Abdominal examination pinpointed abdominal distension and her discomfort during palpation was evident through facial expressions. There was a tympanic sound upon percussion of the abdomen, and bowel sounds were actively heard. Due to her uncooperativeness, a rectal examination couldn't be conducted.

Her lab results highlighted hypokalemia with a potassium level of 2.4 mEq/L. Her creatinine and other electrolytes like sodium, magnesium, and calcium were within the normal range. A complete blood count revealed thrombocytosis with a platelet count of 675 x 10^9/L and leukocytosis with a white blood cell count of 15.6 x 10^9/L. Chronic diarrhea tests, including thyroid stimulating hormone (TSH), celiac panel, cortisol levels, and glycated hemoglobin (Hb1Ac), were unremarkable, as was the stool analysis.

**Figure 1:**
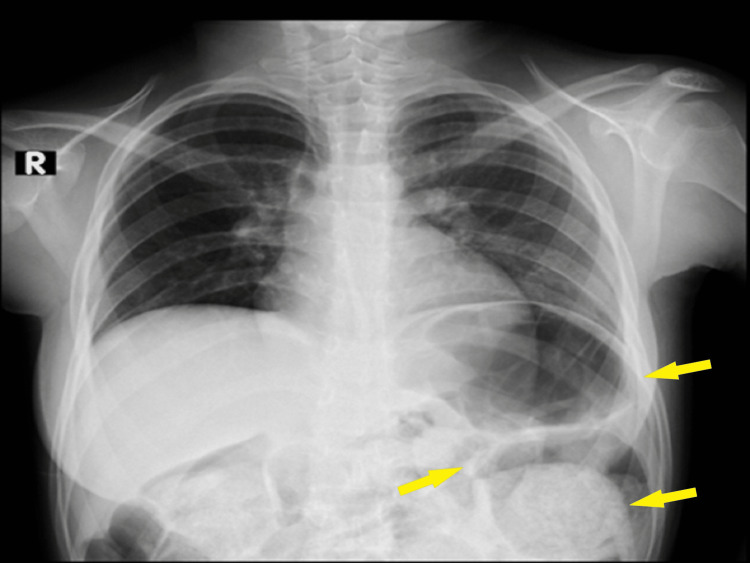
The patient's chest X-ray Yellow arrows show a dilated bowel pushing the diaphragm superiorly, creating a mass effect on the left side.

After being started on trimethoprim/sulfamethoxazole and potassium replacement, imaging was crucial in assessing her condition. A chest X-ray illustrated a dilated bowel pushing the diaphragm superiorly, causing a mass effect on the left side (Figure 1).

**Figure 2:**
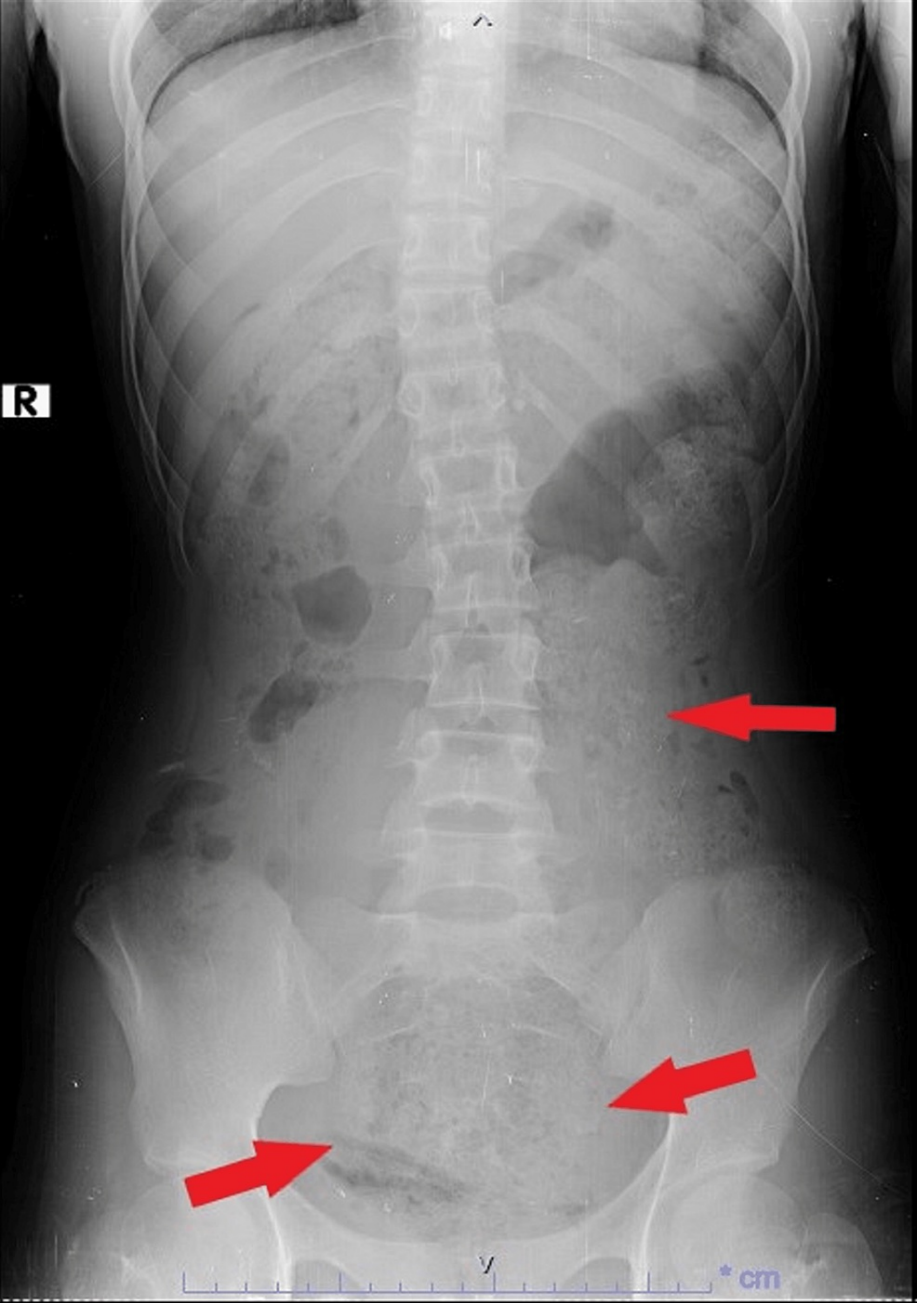
The patient's abdominal X-ray Red arrows reveal a loaded colon and an apparently loaded rectum.

An abdominal X-ray displayed a loaded colon, notably an apparently loaded rectum (Figure 2).

**Figure 3:**
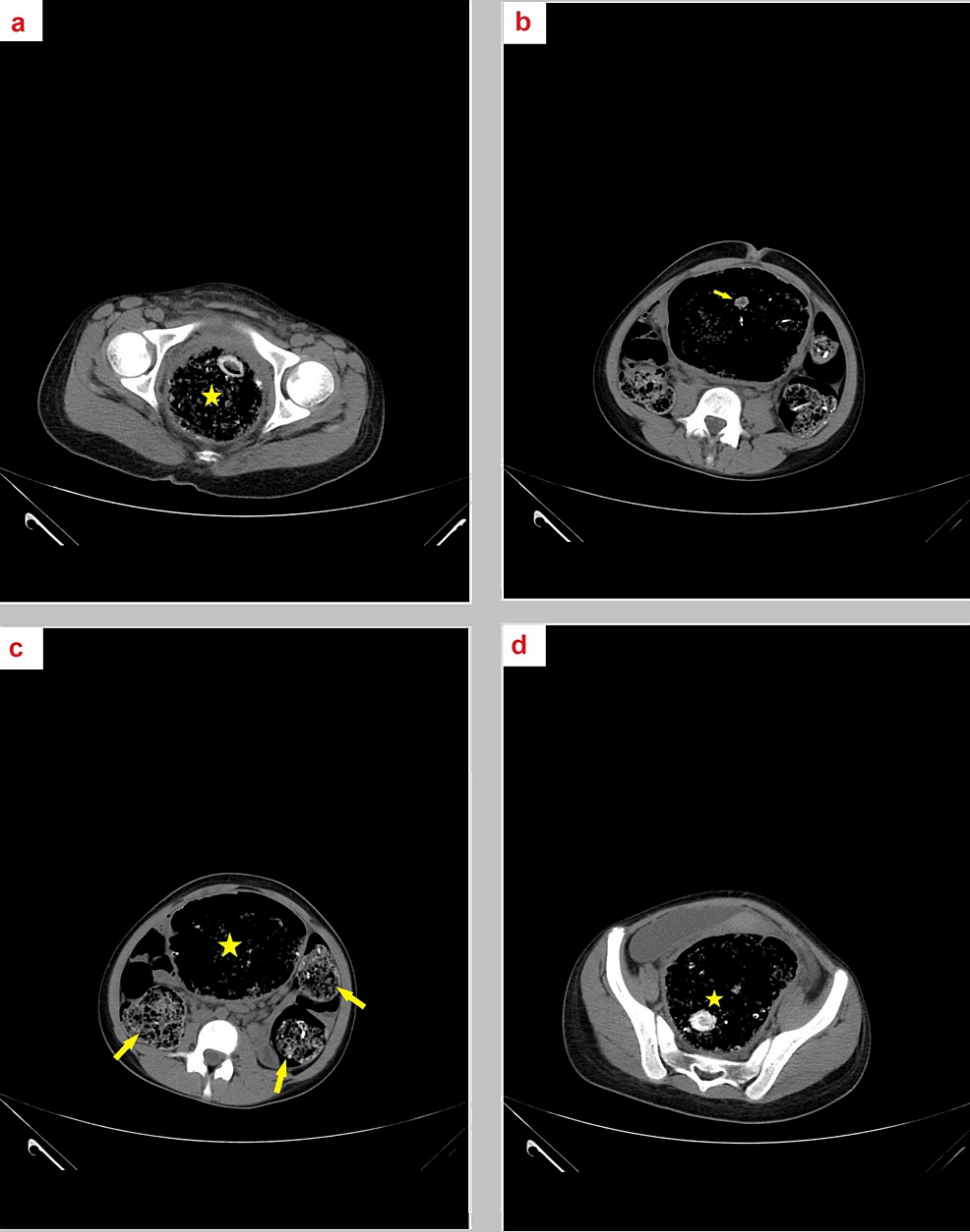
Computed tomography of the abdomen and pelvis (CTAP) displays a dilated large bowel loop with bizarre material present with a severely dilated colon, causing a mass effect on the small bowel loop and the urinary bladder (a-d)

A CT scan depicted a dilated, large bowel loop containing bizarre material. The severely dilated colon exerted a mass effect on the adjacent small bowel loops and the urinary bladder without signs of perforation (Figure 3).

**Figure 4:**
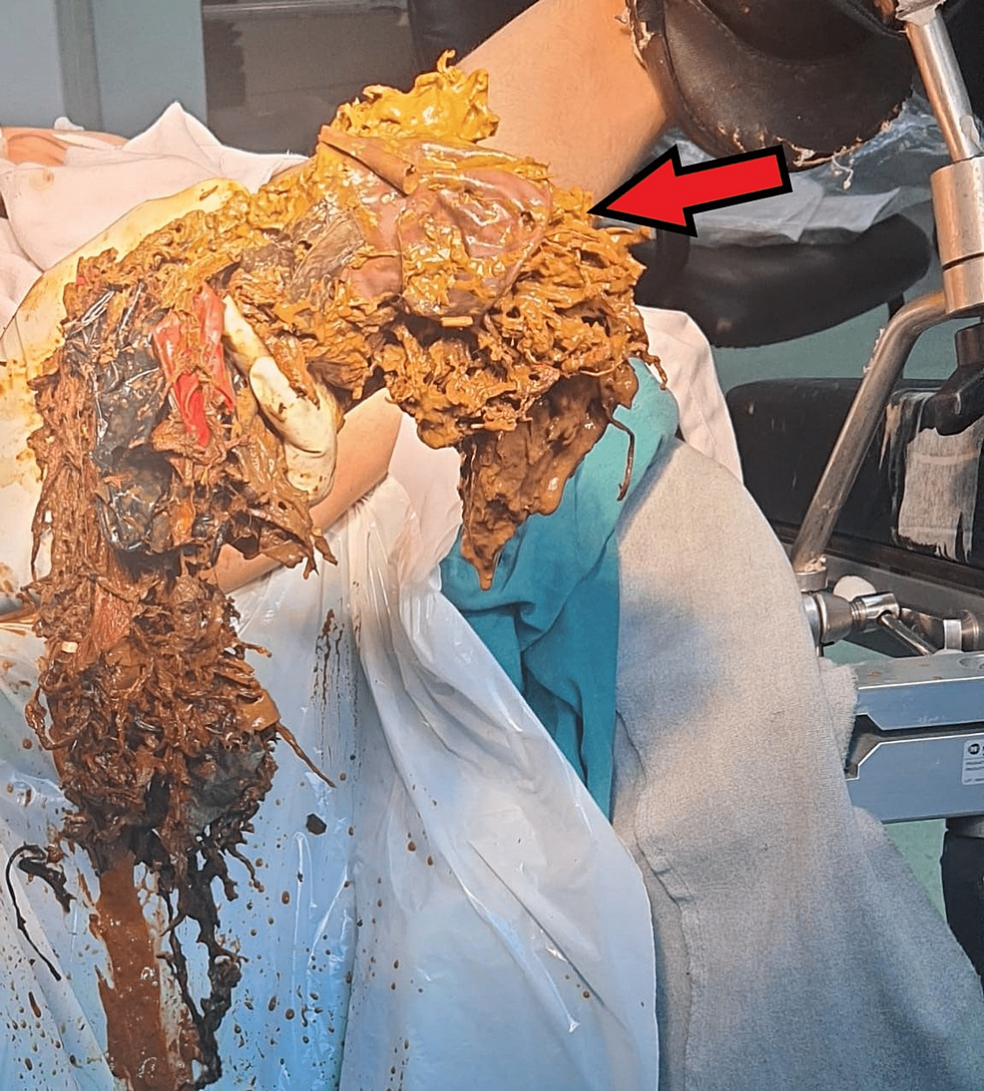
Evacuation under anesthesia: extraction of rectal bezoars The image shows the removal of rectal bezoars, comprising an assortment of unexpected items such as hairs, balloons, plastic bags, threads, and cables, in addition to stool.

This warranted the consultation of a surgical team, which decided on an evacuation under anesthesia (EUA). The procedure entailed a gentle removal of rectal bezoars, which included surprising items like hair, balloons, plastic bags, threads, and cables, along with stool (Figure 4).

Post-procedure, her abdominal distension subsided, and her pain was visibly alleviated. She was discharged the next day with a prescription for polyethylene glycol and instructions for three sachets for one day. A follow-up appointment with the surgeon was scheduled for one week later, but the patient did not return for the follow-up.

Further insight into her personal background revealed that she lived with her father and an elderly grandmother with limited self-care capabilities due to her age. The parents were divorced. There was an apparent underlying neglect regarding the patient, given the lack of treatment for her autism and no follow-up for her gastrointestinal issues. In light of these observations, her case was escalated to the Child Protection Committee at the hospital to ensure her safety and well-being.

## Discussion

Abdominal pain, as evidenced by our 14-year-old patient, is a frequent complaint in the pediatric population. In this particular case, the distress was notably accompanied by chronic diarrhea and lower abdominal tenderness. A comprehensive evaluation, including multiple lab tests and a computed tomography imaging of the abdomen and pelvis (CTAP) scan, ultimately identified fecal impaction and the presence of various rectal bezoars. Interestingly, trichobezoars, which our patient had in the form of a tuft of hair, among other items, are an exceptionally rare cause of abdominal pain, accounting for less than 1% of pediatric cases [9].

Trichobezoars are primarily seen in young female patients, a demographic our patient fits into. It is significant that many of these individuals have coexisting psychiatric disorders. Conditions such as trichotillomania, where an individual compulsively pulls out their hair, and trichophagia, where they consume the hair, are often correlated. Our patient's diagnosis of autism and the anecdotal connections between psychiatric conditions and histories of neglect or abuse further emphasize the necessity of considering such rare causes in specific patient profiles [10-11].

A rarer manifestation, known as Rapunzel syndrome, is when the bezoar extends from the stomach into the duodenum or even further [12]. Though our patient did not exhibit this particular syndrome, the presence of various bezoars highlights the need for high clinical suspicion and thorough evaluation. Imaging is of paramount importance in these scenarios. In our patient's case, the CTAP played a pivotal role in identifying the extent of the bezoar and the associated fecal impaction. Plain radiographs might detect obstructions but have limited success in pinpointing bezoars, with success rates between 10% and 18% [13-14]. However, CTAP, as used in our evaluation, showcases a staggering 97% success rate, offering detailed insights into the obstruction's size, location, and cause [2].

The gold standard for diagnosing trichobezoars remains the upper gastrointestinal endoscopy [15], although our patient's treatment was largely surgical, driven by the size and nature of the bezoar. This deviation from the endoscopy approach is in line with recommendations that larger bezoars warrant an exploratory laparotomy [16].

Revisiting our patient's history and the familial and environmental conditions she was subjected to (living with an elderly grandmother and a father with underlying elements of neglect) play an integral role in understanding and managing such cases. The potential for recurrence is evident, given the patient's social situation and her absence from the follow-up appointment. It is imperative, as suggested, to include biannual abdominal imaging and routine checks for such patients [17]. Moreover, addressing underlying psychological and environmental factors, as highlighted by the involvement of the Child Protection Committee in our patient's case, is essential for comprehensive care and to mitigate the risk of recurrence.

Our case of a 14-year-old female underscores the importance of maintaining a broad differential diagnosis, especially in patients with coexisting psychiatric conditions or histories of neglect. Moreover, it reaffirms the significance of a multidisciplinary approach (surgical and social management) in ensuring the well-being of such vulnerable individuals.

## Conclusions

The intricate presentation of a 14-year-old autistic female with bezoars underscores the multifaceted nature of such cases, often intertwined with psychosocial dynamics. The presence of trichobezoars and non-biological materials in this patient accentuates the need for high clinical vigilance, especially when accompanying psychiatric or socio-environmental challenges are evident. This case emphasizes the importance of a multidisciplinary approach in diagnosis, intervention, and long-term management, ensuring holistic well-being for patients, especially those with underlying conditions like autism. It also highlights the value of integrating clinical care with Child Protection Services when neglect or potential harm is suspected.
